# Scientific Nursing

**Published:** 1873-06

**Authors:** 


### SCIENTIFIC NURSING.

About one-half of all the pretty little babies we see, never reach the age of seven years; one out of four dies before it is a year old. A good deal of forty-rate wit is at the expense of the doctors; they kill more than they cure, and all that. The family of the noble Queen of England consisted of nine children, who have all grown to maturity under the supervision of educated allopathic physicians, who laid down certain principles of management in reference to food, exercise, out-door air, dress, studies, and work. An example of what may be done nearer home, is found in the report of the Visiting Committee of the New York Juvenile Asylum, at Washington Heights; this committee makes a weekly report. The house of reception is in Thirteenth Street, New York. This institution has been in operation twenty-one years, averaging now six hundred persons under its charge all the time, and yet only sixty-seven deaths have taken place. In the healthiest communities which have given reports, the death-rate is twelve out of every thousand each year; in New York city, about thirty die out of each thousand annually. Suppose the 16,454 were for one year, the death rate at twelve would be one hundred and ninety seven persons; at thirty, it would reach nearly five hundred instead of sixty-seven; this is merely an approximation. As far as life-saving is concerned, it would be a good thing if the children of those who have grown up and married in perfect ignorance of everything pertaining to the proper management of infancy and childhood were sent to the Juvenile Asylum, where they would be under scientific management. Surely some sort of information pertaining to the duties of motherhood ought to be imparted to the daughters of the land, before they are assumed.

